# Psychometric properties of the Arabic translation of the Dark Future Scale questionnaire in a non-clinical sample of Arabic-speaking young adults

**DOI:** 10.1186/s12888-024-05822-z

**Published:** 2024-06-18

**Authors:** Joya-Maria Karam, Diana Malaeb, Rabih Hallit, Feten Fekih-Romdhane, Sahar Obeid, Souheil Hallit

**Affiliations:** 1 Faculty of Medicine, Lebanese University, Beirut, Lebanon; 2 College of Pharmacy, Gulf Medical University, Ajman, United Arab Emirates; 3 School of Medicine and Medical Sciences, Holy Spirit University of Kaslik, P.O. Box 446, Jounieh, Lebanon; 4 Department of Infectious Disease, Notre Dame, Secours University Hospital Center, Street 93, Byblos, Postal Code 3, Byblos, Lebanon; 5 Department of Infectious Disease, Bellevue Medical Center, Mansourieh, Lebanon; 6 The Tunisian Center of Early Intervention in Psychosis, Department of Psychiatry “Ibn Omrane”, Razi hospital, Manouba, 2010 Tunisia; 7 Faculty of Medicine of Tunis, Tunis El Manar University, Tunis, Tunisia; 8 Social and Education Sciences Department, School of Arts and Sciences, Lebanese American University, Jbeil, Lebanon; 9 Psychology Department, College of Humanities, Effat University, Jeddah, 21478 Saudi Arabia; 10 Applied Science Research Center, Applied Science Private University, Amman, Jordan

**Keywords:** Anxiety, Future anxiety, Psychometric properties, Non-clinical sample, Scale validation, Confirmatory factor analysis, Arabic

## Abstract

**Background:**

Through the years, studying negative behaviors of the worldwide population seized the spotlight from many researchers who focused on building scales in order the measure the level of worries, fear and even depression of such stressed individuals. By definition, “Future anxiety” (FA) is fueled by negative thoughts leading to intense fear of unknown future events. The Dark Future scale (DFS) measures the level of anxiety experienced towards the future. Our aim was to examine the psychometric properties of a novel Arabic translation of the DFS.

**Methods:**

A sample of 684 Arabic-speaking young adults (65.6% women) filled the DFS, TEMPS-M (temperaments) and DASS-8 (psychological distress).

**Results:**

Confirmatory factor analyses (CFA) supported a unidimensional model of the DFS score, with all 5 items retained. This scale had good reliability. Moreover, concurrent validity demonstrated significant associations between DFS scores and psychological distress, depressive, cyclothymic, irritable and anxious temperament. Scores achieved scalar invariance across gender, with women having greater exposure to anxiety about the future.

**Conclusion:**

Overall, these findings led to the conclusion that the Arabic DFS is a psychometrically valid tool for the assessment of FA. The DFS is a brief, reliable and easy to apply scale that would help researchers in psychology and psychiatry in assessing anxiety about future.

## Introduction

The human mind has been found to work as an “anticipatory machine” by creating images and scenarios for the future in order to predict upcoming events based on previous positive or negative experiences [1, 2]. The intention of “anticipating the future” too rigidly with unrealistic expectations can lead to anxiety and disappointment if things do not unfold as expected. This process of constant fear would contribute to high levels of stress and anxiety [1]. In 1996, the expression “future anxiety” was first introduced by Zaleski [3], which refers to a pessimistic outlook of the future where negative thoughts overshadow positive ones, leading to an overwhelming negativity [4]. The attitude of human beings toward the future varies greatly, ranging from optimism to pessimism. Therefore, several studies [4, 5] explored how personality traits can affect the ability to deal with threats and decide if forthcoming events will be faced with worry, fear and stress. Moreover, the environment and current situation of a person (economic status, a disabled family member…) have been shown to affect their perception of the future; for instance, in Iran, a recent study [6] conducted among mothers with disabled children concluded that they experience higher levels of anxiety about the future, health, and welfare. In addition, they tend to adopt a negative mindset: pessimism, helplessness and avoidance of social relations, which means that negative experiences can predispose the individual to depression and negative emotions. Moreover, in China, a research conducted among parents with autistic kids shows that elevated parenting stress accompanied with financial troubles would make them less hopeful about their capabilities to sustain a decent future for their families [7]. Worldwide, the buildup of stress among anxious parents due to concern about their children’s future must be quantified to evaluate the psychological impact on different populations.

A study comparing two Arabic-speaking countries (Lebanon and Qatar) with USA reported higher levels of depression and anxiety among college student in Qatar and Lebanon compared to the USA, which gives us additional reasons to replicate an Arabic version of the DFS [8].

Until now, many researchers have driven the further development of scales to assess anxiety and stress. To measure “future anxiety”, Zaleski et al. [3] developed the Dark Future Scale (DFS). Beginning with a pool of 29 items, the DFS has been reduced to 10 items by performing an exploratory factor analysis (EFA) [9, 10]. A total of 14 items have been removed because of low loading scores, leading to five remaining items that are considered the most representative to measure pessimism about the future. A confirmatory factor analysis (CFA) conducted on the final scale a second study showed good psychometric properties [4]. Moreover, the test-retest reliability over a one-month interval was satisfying.

The psychometric properties of the DFS have also been examined in various national and cultural contexts; validation studies using this scale among adults from Iran [6], Spain [11], Italy [12] and Turkey [13] showed adequate coefficients of reliability (0.7, 0.7, 0.85 and 0.86 respectively). Good internal consistency (= 0.77)was found in another study conducted among children and adolescents aged 8 to 18 years in Germany to assess the aftermath of COVID-19 [14].

As a contribution to this pool of global research, the present study examined the psychometric properties of an Arabic version of the DFS questionnaire in a non-clinical sample of Arabic speaking young adults. We found that introducing an Arabic version of the DFS being useful for multiple reasons. First, no previous research has investigated the DFS in the Arab region although it has been found to have the highest level of anxiety disorders especially Arab countries involved in tensions, political conflicts, financial instabilities such as Iraq, Lebanon and Afghanistan [15]. Second, Arabic language is widely used when conducting research in the mental health field [16]. It is now spoken in 25 countries with 30% of foreigners that speak Arabic are in the Western countries [17].

In the Arab world, multiple studies reported that the median age onset of anxiety disorders is in the early to late teens [18, 19]. In addition, another study conducted among 1552 adolescents in Arab countries (Abha city, southwestern Saudi Arabia) showed that mental disorders amounted to 15.5% from which anxiety was the most prevalent finding specifically in Arab-speaking countries with a history of war, conflicts and economic instabilities [20]. In literature, numerous studies tried searching for different factors that would trigger anxiety in this age group; neglect, child abuse, financial and cultural instability were shown as correlates of anxiety among Lebanese young adults [21]. Arab American college students were at increased risk for poor mental health and future anxiety relative to their non-Arab American peers; this was speculated to be due factors related to religiosity and discrimination following their cultural and traditional practices as Arabs [22].

Most importantly, a global emergence of a novel concern has emerged among young adults, the international migration for higher education, which exposes them to high levels of uncertainty and doubt towards the future [23]. Aiming at examining the level of future anxiety among international students originating from war and conflict areas such as Yemen (where Arabic is the official spoken language), a recent investigation explained that these individuals live in constant fear of future events [3], threatening their education, profession, carrier, financial and social situation because of their previous traumas (conflicts and instabilities) in their native country. Moreover, it has been found that young people from the Middle East were subject to racism and discrimination in their host countries [24].

### The present study

The aim of this study was to translate the DFS to Arabic and examine its psychometric properties (reliability, concurrent and construct validity) among Arabic speaking young adults, and measurement invariance between genders. Given that both the original development study [3] and all subsequent test adaptation studies (e.g [4, 13]. . , have supported a unidimensional model of DFS with all 5 items retained, we expected to find similar evidence here. In addition, we excepted that this model would have invariance across women and men, which would be consistent with previous work [4], and have adequate concurrent validity with psychological distress and temperaments. For instance, a previous study [5] conducted in Lebanon suggested that experiencing psychological distress is a moderator for the relationship between negative temperament (anxiety and fear) and dark future.

## Methods

### Procedures

Data for this cross-sectional study was collected via a Google Form link, between February and March 2023. The research team approached people and asked them to fill the survey; those who accepted were asked to forward the link to other people they might know, explaining the snowball sampling technique followed. Inclusion criteria for participation included being of a resident and citizen of Lebanon of adult age. Excluded were those who refused to fill out the questionnaire. After providing digital informed consent, participants were asked to complete the instruments described above, which were presented in a pre-randomized order to control for order effects. The survey was anonymous and participants completed the survey voluntarily and without remuneration [25].

### Measures

#### Dark future scale

Participants filled a novel version of an Arabic translation of the 5-item DFS [4]. All items were rated on a 7-point scale from 0 (Decidedly false) to 7 (Decidedly true). Before their use in the current study, the DFS scale was translated and adapted to the Arabic language and context. To this end, it was translated to the Arabic language with the purpose of achieving semantic equivalence between measures in their original and Arabic versions following international norms and recommendations [26]. For this, the forward and backward translation method was applied. The English version was translated to Arabic by a Lebanese translator who was completely unrelated to the study. Afterwards, a Lebanese psychologist with a full working proficiency in English, translated the Arabic version back to English. The translation team ensured that any specific and/or literal translation was balanced. The initial and translated English versions were compared to detect/eliminate any inconsistencies and guarantee the accuracy of the translation by a committee of experts composed of two psychiatrists and one psychologist, in addition to the research team and the two translators [27]. An adaptation of the measure to our specific context was performed, and sought to determine any misunderstanding of the items wording as well as the ease of items interpretation, and therefore, ensure the conceptual equivalence of the original and Arabic scales in both contexts [28]. After the translation and adaptation of the scale, a pilot study was done on 30 patients to ensure all questions were well understood; no changes were applied after the pilot study.

#### TEMPS-M

Participants were asked to complete the Temperament Evaluation in Memphis, Pisa and San Diego (TEMPS-M), validated in Arabic [29]. This scale consists of 35-item scored on a 5-point Likert scale ranging from 1 (not at all) to 5 (very much). The factor analysis confirmed five dimensions [30]: (1) Depressive temperament (i.e., susceptibility to feel sad and to be more emotionally sensitive) (ω = 0.87 / α = 0.87) (2) Cyclothymic temperament (i.e., experiencing cyclic mood fluctuations) (ω = 0.89 / α = 0.89) (3) Hyperthymic temperament (i.e., experiencing elevated or positive mood with high energy levels) (ω = 0.85 / α = 0.85) (4) Irritable temperament (i.e., susceptibility to express frustration more easily than the average person) (ω = 0.85 / α = 0.85) (5) Anxious temperament (i.e., tending towards worry, ruminate, and continuous tension) (ω = 0.87 / α = 0.87). Subscale scores range from 5 to 35, with higher scores denoting a higher expression of the temperament.

#### Depression, anxiety and stress 8 items (DASS-8)

Participants were asked to complete the DASS-8 items, validated in Arabic [31]. The questions are rated on a 4-point Likert scale (“0 = does not apply to me” to “3 = always applies to me”). Higher scores reflect more psychological distress. The DASS-8 expressed excellent internal consistency (ω = 0.90 / α = 0.90).

#### Demographics

Participants were asked to provide their demographic details consisting of age, sex, and highest level of education.

### Analytic strategy

#### Confirmatory factor analysis (CFA)

There were no missing responses in the dataset. We used data from the total sample to conduct a CFA using the SPSS AMOS v.29 software. The minimum sample size to conduct a CFA was 100 participants based on 20 times the number of the scale’s variables [32]. Parameter estimates were obtained using the maximum likelihood method. Fit indices, such as the normed model chi-square (χ²/df), the Steiger-Lind root mean square error of approximation (RMSEA), the standardized root mean square residual (SRMR), the Tucker-Lewis Index (TLI) and the comparative fit index (CFI), were computed. Values ≤ 5 for χ²/df, ≤ 0.08 for RMSEA, ≤ 0.05 for SRMR and ≥ 0.90 for CFI and TLI indicate good fit of the model to the data [33]. Additionally, evidence of convergent validity was assessed with average variance extracted (AVE) values of ≥ 0.50 considered adequate. Multivariate normality was not used at first (Bollen-Stine bootstrap *p* = .024 < .05); therefore, we performed non-parametric bootstrapping procedure.

#### Gender invariance

To examine gender invariance of DFS scores, we conducted multi-group CFA using the total sample [34]. Measurement invariance was assessed at the configural, metric, and scalar levels [35]. We accepted ΔCFI ≤ 0.010 and ΔRMSEA ≤ 0.015 or ΔSRMR ≤ 0.010 (0.030 for factorial invariance) as evidence of invariance [36].

#### Reliability and concurrent validity

Composite reliability was assessed using McDonald’s ω and Cronbach’s α, with values greater than 0.70 reflecting adequate composite reliability [37]. The skewness and kurtosis values varied between − 1 and + 1 for the DFS score [38]. Pearson test was used to correlate the DFS scores with the other scales in the survey. Student t test was used to compare two means. According to Cohen, correlation coefficients values ≤ 0.10 were considered weak, ~ 0.30 were considered moderate, and ~ 0.50 were considered strong correlations. *P* < .05 was deemed statistically significant.

## Results

### Participants

Six hundred eighty four young adults filled the survey, with 65.6% females and a mean age of 21.74 ± 4.30 years. Sample’s details are summarized in Table 1.

**Table 1:** Sample’s characteristics by gender

	Total (*n* = 684)	Men (*n* = 235)	Women (*n* = 449)
Gender			
Men	235 (34.4%)		
Women	449 (65.6%)		
Education			
Secondary or less	13 (1.9%)	9 (3.8%)	4 (0.9%)
University	671 (98.1%)	226 (96.2%)	445 (99.1%)
Age (years)	21.74 ± 4.30	22.07 ± 4.43	21.56 ± 4.22

### Confirmatory factor analysis of the DFS scale

**Fig. 1:**
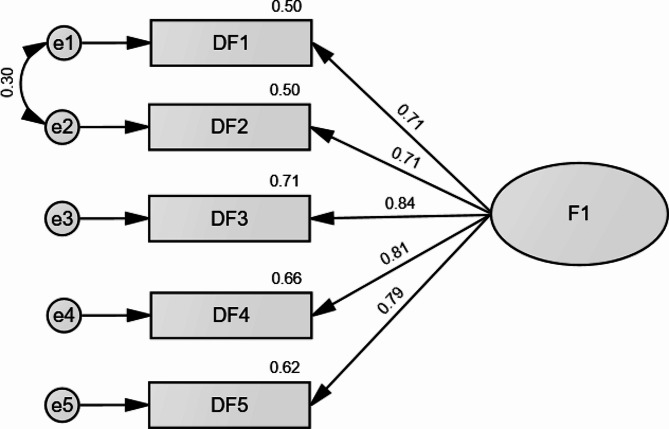
Standardized loading factors of the Dark Future Scale items in Arabic

There was an absence of multicollinearity through variance inflation factor (VIF) values < 5. CFA indicated that the fit of the one-factor model of the DFS scale was acceptable: χ^2^/df = 63.46/5 = 12.69, RMSEA = 0.131 (90% CI 0.103, 0.160), SRMR = 0.034, CFI = 0.968, TLI = 0.935. We added a correlation between residuals of items 1 and 2 due to high modification indices; the results improved as follows: χ^2^/df = 17.79/4 = 4.45, RMSEA = 0.071 (90% CI 0.040, 0.106), SRMR = 0.016, CFI = 0.992, TLI = 0.981. The standardized estimates of factor loadings were all adequate (Fig. 1). The convergent validity for this model was very good, as AVE = 0.77.

### Composite reliability

Composite reliability of scores was adequate in the total sample (ω = 0.89 / α = 0.89), males (ω = 0.88 / α = 0.88), and females (ω = 0.89 / α = 0.89).

### Gender invariance

As reported in Table 2, we were able to show the invariance across gender at the configural, metric, and scalar levels. A significantly higher mean dark future score was seen in females compared to males (18.67 ± 7.71 vs. 16.49 ± 8.03; *t* = -3.457; *p* = .001).

### Concurrent validity

Higher DFS scores was moderately to strongly associated with more psychological distress (*r* = .42; *p* < .001), depressive (*r* = .48; *p* < .001), cyclothymic (*r* = .51; *p* < .001), irritable (*r* = .20; *p* < .001) and anxious (*r* = .40; *p* < .001) temperaments (Table 3).

**Table 2:** Measurement invariance of the dark future scale across gender in the total sample

Model	CFI	RMSEA	SRMR	Model Comparison	ΔCFI	ΔRMSEA	ΔSRMR
Configural	**0.967**	0.093	0.039				
Metric	**0.965**	0.082	0.044	Configural vs. metric	0.002	0.011	0.005
Scalar	**0.966**	0.070	0.044	Metric vs. scalar	0.001	0.012	< 0.001

*Note.* CFI = Comparative fit index; RMSEA = Steiger-Lind Root Mean Square Error of Approximation; SRMR = Standardised root mean square residual.

**Table 3:** Correlation matrix of the continuous variables

	1	2	3	4	5	6	7
1. Dark Future	1						
2. Psychological distress	0.42***	1					
3. Depressive temperament	0.48***	0.68***	1				
4. Cyclothymic temperament	0.51***	0.67***	0.71***	1			
5. Hyperthymic temperament	0.004	− 0.11**	− 0.08*	0.03	1		
6. Irritable temperament	0.20***	0.47***	0.55***	0.48***	0.08*	1	
7. Anxious temperament	0.40***	0.64***	0.57***	0.59***	0.06	0.43***	1

## Discussion

Research on Dark future or “future anxiety” has raised interest in scholars to develop and validate a scale that measure the degree of anxiety in anticipation of the future [4, 12]. The DFS has been shown to be a valid and reliable instrument in a wide range of international contexts [7, 12, 13]. As a contribution to this literature, we examined the psychometric properties of an Arabic version of the DFS. Our results supported the unidimensional structure of the DFS, as well as its invariance across gender, high composite reliability and good concurrent validity.

In terms of the factorial validity of the Arabic DFS, our results are consistent with previous work showing that all 5 items were retained and loaded on one factor [4]. Based on the CFA, our results suggest good fit indices and strong loading factors (> 0.7) on the single latent factor (Dark future) with the observed variables measuring a common underlying construct [39]. This would suggest that the items chosen are significant indicators of individuals’ perceptions of a negative future.

Furthermore, the internal reliability of the Arabic DFS was high as shown by high omega and alpha values (= 0.89). From these results, it is clear that our items are closely related and can are able to measure the same construct with reliability [40]. These findings are comparable to the findings reported by Zaleski et al. [4] (α = 0.92) but higher than the Turkish version of the DFS (α = 0.79) [41].

Our findings also indicated that the unidimensional factor structure of DFS scores was invariant between genders, with higher scores seen in females. These findings are in accordance with findings reported by a systemic review [42] including 44 articles showing that women have higher risk of future anxiety than man and this has been repeatedly discussed to be due to hormonal fluctuations [43], interpersonal relationships, rumination and worry more prominent in women [4, 42, 44]. Women of the Middle East and Northi Africa (MENA) region are under an overwhelming pressure [45] stemming from sociocultural norms regarding gender roles placing significant stress on women to conform to societal standard and their dual role as the center of the family and their participation in the workforce [46, 47]. We can also mention that women in the MENA region are often deeply concerned about their children’s future due to ongoing geopolitical conflicts and instability. This has been introduced as a “silent epidemic of depression” experienced by Arab women [48]. It has been agreed that cultural norms related to education, career, family expectations, marriage and the well-being of their children are key factors contributing to this “epidemic” [49]. In the case of our study, it is difficult to specify the exact cause of this gender difference in the absence of additional data A field of upcoming investigations in the Arab World would be of great significance to provide deeper explanation about the different triggers affecting genders in order to anticipate the future and perceive it as “dark”.

Regarding concurrent validity, our results showed a significant association between higher DFS scores and higher psychological distress. A similar conclusion has been reached by a previous experiment conducted in Yemen among a sample of similar age (19–30 years) [24], featured by the fear of failure in their academic path and the fear of lack of job opportunities, which threatens their future and their financial and social security. It has been clearly substantiated that worrying about upcoming events will negatively affect young adults’ perception, ability to concentrate, interactions and performance, making them less determined towards their goals and ambitions. Other researchers [50] explain this as a “stage” in young adults’ life called identity recognition, experienced due to heavy social demands to build a robust future without any prior orientation, resulting in severe anxiety. The same study has proposed an awareness program that replaces “future anxiety” by “future orientation” to help young people anticipate their future in a healthier way.

Furthermore, our investigation reported a positive correlation between future anxiety and depressive, irritable, cyclothymic and anxious temperaments. The individual would be at risk to be crippled by negative emotions, feeling of hopelessness and dissatisfied with his/her life. Unfortunately, multiple explorations [51–53] of uncontrolled cyclothymic temperaments predicts high risk of suicidal ideation due to a hopelessness regarding their future originating from their anxiety and negative thoughts.

### Clinical implications

Besides helping clinical scientists in their research settings, and pending future validation studies in clinical settings, the DFS can be applied to people with psychosomatic problems and those confronted with stressful life events (such as patients with depression, those undergoing a major surgery, patients with chronic illnesses, or even individuals who are experiencing a life transition such as divorce or job loss). Assessing very high levels of stress and anxiety about the future is necessary for successful psychological work that will aim, first, at reducing the paralyzing fear connected to the future.

### Limitations

As regards to the limitations of our research, it can be mentioned that the sample used was only limited to a certain age group (young adults with a mean age of 21.74 years). Moreover, we note that psychometric instruments used in this study were related to a non-clinical sample of Arabic speaking population. Information bias is possible since answers were self-reported. Other psychometric properties are missing in this paper (test-retest). Furthermore, our sample is gender-skewed, in favor of females (449 females vs. 235 males). Although the findings of this study corroborate the ones of previous validation papers, a selection bias is present since the sample was collected in a convenient way. We should also note that the sample used in our study included majorly individuals with higher levels of education; these individuals may possess different cognitive abilities, coping mechanisms and socio-economic background compared to the general population. Finally, future studies should investigate the invariance of the DFS across different linguistic groups within and between countries and cultures [11]. Besides, the Arabic version of the DFS should be tested in other Arab countries, and in clinical samples. Since the sample was recruited conveniently and is composed of a majority of females and participants of a university level of education, results might not be representative of the whole population.

## Conclusion

This study conducted among a non-clinical sample has successfully validated the DFS in its Arabic version. It is a valid instrument to be used in upcoming investigations among young adults’ samples. This scale will help assess future anxiety and the level of worry an individual holds towards his/her future. The availability of the Arabic DFS may help public health practitioners better understand the level of anxiety crippling young adults in their academic paths, social lives and personal lives.

## Data Availability

All data generated or analyzed during this study are not publicly available due the restrictions from the ethics committee, but are available upon a reasonable request from the corresponding author (SH).
